# Ethnic Enclaves, Self-employment, and the Economic Performance of Refugees: Evidence from a Swedish Dispersal Policy

**DOI:** 10.1177/0197918320912195

**Published:** 2020-05-05

**Authors:** Henrik Andersson

**Affiliations:** 370111 Uppsala University, Uppsala

**Keywords:** Immigration, self-employment, ethnic enclaves, co-ethnics

## Abstract

This article estimates the causal effect of residential concentration of co-ethnics (ethnic enclaves) on the probability to start a business among refugees in Sweden. Results indicate that the share of self-employed co-ethnics in the port of entry municipality increases refugees’ probability of entry into self-employment, while the actual share of local co-ethnics has no effect or, in some cases, a negative effect. The results support the conclusion that skills and resources within the local ethnic enclave, particularly skills relevant for self-employment, are crucial for generating new entry into self-employment for refugees, while simply more co-ethnics, plausibly increasing an ethnic market’s size, are of less importance. Moreover, the results suggest that being placed with a larger share of self-employed co-ethnics is negatively related to refugees’ long-term disposable income; however, assuming there is no or little selection of high-ability refugees into self-employment, this negative relationship can be counteracted by the choice of self-employment. The study adds new knowledge on the arguably crucial topic of socio-economic integration of an important group of international migrants — namely, refugees.

## 1. Introduction

The residential segregation of foreign-born residents presents an interesting policy trade-off. On the one hand, social and physical distance from native-born residents can decrease their access to host-country skills (Danzer and Yaman 2016). On the other hand, residential concentration of co-ethnics^1^ (ethnic enclaves) can foster networking and, thereby, employment opportunities for immigrants (Edin, Fredriksson, and Åslund 2003). Understanding the relationship between enclaves and economic activity is, therefore, important for assessing the impact of residential segregation on immigrants’ socio-economic outcome.

Indeed, a fairly large amount of the literature has sought to use various natural experiments to understand and identify ethnic enclaves’ effect on socio-economic outcomes among immigrants. Studies in Danish, Swedish, and US settings have all found positive effects of larger co-ethnic networks on immigrant income and employment (Edin, Fredriksson, and Åslund 2003; Munshi 2003; Damm 2009, 2014; Beaman 2012), as well as on welfare uptake (Bertrand, Luttmer, and Mullainathan 2000; Åslund and Fredriksson 2009). This article, however, attempts to shed further light on the relationship between ethnic enclaves and economic outcomes by estimating the causal effect of residential co-ethnic concentration on the probability of *entry into self-employment* among refugees in Sweden.

Self-employment has particular importance in this discussion, partly because it tends to be more common among foreign-born than native-born residents (OECD 2017) and partly because previous research from Sweden has shown a tendency of immigrant managers to hire other co-ethnics (Åslund, Hensvik, and Nordström-Skans 2014). While previous observational studies have examined the relationship between an enclave’s size and the probability of self-employment among immigrants (e.g., Borjas 1986; Yuengert 1995; Le 2000; Clark and Drinkwater 2002, 2010; Andersson and Hammarstedt 2015), quasi-experimental evidence remains rare. Exceptions include Eliasson (2014), who found a positive effect of co-ethnic *bankers* on the probability of self-employment among immigrants, and Damm (2009), who used Danish data to study the effect of the local ethnic enclave’s size on refugees’ economic performance, finding no effect on self-employment. Overall, however, there is a great need for more causal evidence on the relationship between ethnic enclaves and self-employment and, thus, more research using quasi-experimental design.^2^

In addition to providing a sound research design, I also attempt to separate between relevant mechanisms. Broadly speaking, there are at least two ways in which the concentration of co-ethnics and self-employment are connected (e.g., Light 1984; Borjas 1986). First, self-employment can be a function of the *quantity* of co-ethnics. A larger number of co-ethnics could imply more networking and a larger potential market. Entrepreneurs can sell to these niche (ethnic) markets, which, in turn, also provide employment (Borjas 1986). Second, individuals with particular qualities or *resources* (Light 1984) can provide co-ethnics with know-how, skills, contacts, and capital, all of which are useful elements to set up businesses. In this article, I attempt not only to estimate the average effect of ethnic enclaves on refugees’ entry into self-employment but also to address the aforementioned mechanisms (quantity of co-ethnics and resources within the ethnic enclave).

The setting for this analysis is Sweden, which has a long tradition of refugee intake and, for some time, increasing levels of residential segregation (Åslund and Nordström-Skans 2010). The Swedish context also allows me to capitalize on two key institutional features without which the quasi-experimental research design would not be possible. First, in Sweden, there is high-quality individual information on all residents, which allows me to investigate the probability of self-employment as a function of both detailed individual characteristics and neighborhood resources, especially the share of municipal residents who are co-ethnics or self-employed co-ethnics. Second, between 1985 and 1994, Sweden settled refugees with a dispersal policy that placed all newly arrived refugees in contracted municipalities. As has been argued by several researchers (e.g., Edin, Fredriksson, and Åslund 2003; Åslund and Fredriksson 2009; Eliasson 2014), conditional on individual characteristics, the policy created exogenous geographic variation, allowing the researcher to overcome problems of endogeneity. My analysis, therefore, is based on identifying refugees who arrived (were given residence permits) in Sweden in 1990 and 1991. The empirical estimation’s baseline set-up will be a reduced form estimate, in which I regress an indicator of refugee self-employment within five years after arrival in Sweden on ethnic enclave information in the municipality of arrival, which is the result of the dispersal policy.

This article’s main finding is that refugees who were placed in municipalities with a higher share of *self-employed* co-ethnics also entered self-employment to a higher degree over the coming five years. In the preferred specification, a standard deviation increase in the share of co-ethnics with business income increases the probability of refugees entering self-employment by around two percentage points. Given that only around 4.5 percent of the sample earned any business income within the first five years, this effect is not negligible. The main specification further includes country and municipality fixed effects; hence, the effect is not due to a generally higher occurrence of self-employment in some municipalities or within certain birth countries. On the contrary, I find no effects of the pure number of co-ethnics on refugee entry into self-employment. The estimations, therefore, support a story in which meeting skilled co-ethnics with relevant resources seems to matter greatly for self-employment entry among refugees, while access to more co-ethnics, regardless of skills or abilities, has no effect. A second finding is that being settled with a larger share of self-employed co-ethnics is related to a small long-term negative income development for settled refugees but that this negative relation can be counteracted by the choice to pursue self-employment.^3^

This article adds to several strands of the literature. First, it complements research on the causal effect of ethnic enclaves on immigrants’ economic outcomes (Edin, Fredriksson, and Åslund 2003; Munshi 2003; Damm 2009, 2014; Beaman 2012) by considering self-employment, an outcome that has received very little attention in this literature (Damm 2009; Eliasson 2014). Also, using causal mediation analysis (Imai, Keele, and Tingley 2010), I study the economic impact of ethnic enclaves *and* self-employment by considering refugees’ long-term economic performance. Since it is of interest to consider not only the effect of ethnic enclaves on immigrants’ socio-economic outcomes but also which factors mediate the relationship, causal mediation analysis can provide important complementary evidence to the reduced form results in this study.

Second, I add causal evidence to research demonstrating *associative* evidence regarding the relationship between enclave size and the probability of self-employment among immigrants (e.g., Borjas 1986; Yuengert 1995; Le 2000; Clark and Drinkwater 2002, 2010; Andersson and Hammarstedt 2015). My results are mostly in line with Yuengert (1995), who found no relation between enclave size and immigrant self-employment in the United States, and to some extent with Clark and Drinkwater (2002, 2010), who find worse employment and self-employment outcomes from enclave size among minorities in Britain. On the other end, a positive correlation between enclave size and self-employment is found by a number of studies (Borjas 1986; Le 2000; Lofstrom 2002; Fairlie and Woodruff 2005; Andersson and Hammarstedt 2015). Comparisons to these associative studies should, however, be done with some care, since they do not fully address issues of endogeneity, which implies that results, to a varying degree, may be biased.

Finally, by adding evidence on *one of many* predictors of self-employment entry, this article connects to the broader literature on the determinants of self-employment in general (Simoes, Crespo, and Moreira 2016), as well as to research on business ownership among immigrants and minorities, including variations between ethnic groups (Fairlie and Meyer 1996; Fairlie and Lofstrom 2015). Besides ethnic enclaves, important suggested factors affecting entry into self-employment among minorities include discrimination and marginalization (Constant and Zimmerman 2006; Blume et al. 2009), human capital variation (Lofstrom and Wang 2009), home-country business experience (Akee, Jaeger, and Tatsiramos 2013), and access to capital (Eliasson 2014).^4^

To develop these contributions, this article proceeds with a short theoretical discussion in section 2 on the relation between ethnic enclaves and entry into self-employment. Section 3 introduces the Swedish context, including its refugee placement program, which played a crucial role in the research design. Section 4 provides the empirical details, a description of the data, and sample selection, as well as a more thorough discussion of the empirical model. Finally, results are presented in section 5, followed by a concluding discussion in section 6.

## 2. Mechanisms at Work

As a simple way of conceptualizing the importance of ethnic enclaves in the choice of entry into self-employment, I take my starting point in a Roy-model, which defines the choice of self-employment as a function of the expected outcome of different labor-market options (Roy 1951). In its simplest form, the model implies that any individual chooses self-employment *if* the expected outcome from self-employment is larger than the expected outcome from other accessible labor-market options. If we consider co-ethnics as peers,^5^ they can positively affect relevant local and individual attributes or resources in a number of ways. These attributes in turn may affect the expected self-employment income.

A first mechanism through which this positive relation may occur is via the number of consumers (Borjas 1986). Assuming that people with a specific ethnicity have some common preferences, a local concentration of residents who belong to the same ethnic group can give rise to “ethnic markets.” An ethnically clustered area can create a local demand for different sets of products for which co-ethnics likely hold large knowledge-based comparative advantages. Consumers may also have cultural preferences for dealing with co-ethnics (Aldrich and Waldinger 1990). If self-employed individuals in Sweden open niche-market businesses along ethnic lines, we expect the number of co-ethnics living close by to increase the consumer base.^6^ For this mechanism, the quantity of co-ethnics is the most relevant metric.

A second mechanism through which ethnic enclaves may affect self-employment works through skills needed to start a business in a certain country and place, including institutional and regulatory knowledge, as well as specific self-employment skills. Information sharing within local co-ethnic networks can serve as an important tool to access better knowledge about self-employment procedures (Light 1984). It is reasonable to assume, then, that first and foremost, self-employed co-ethnics, who have themselves gone through the process of opening a business, can inform and instruct newly arrived individuals on self-employment skills. For this mechanism, it is the specific *resources* within the local enclave that matter, rather than its size.

Given the aforementioned mechanisms, it follows that both larger enclaves and enclaves with more relevant resources could increase the incentives to enter self-employment for refugees. However, these mechanisms are simplified, and for a few reasons, enclaves’ effect on self-employment is a priori ambiguous: First, research from Sweden has indicated that immigrant managers tend to hire co-ethnics (Åslund, Hensvik, and Nordström-Skans 2014). Since co-ethnics have a preference for hiring other co-ethnics, a higher share of local residents who are self-employed co-ethnics will increase the chances of regular employment for new immigrants from the same place/background, thereby mechanically lowering the share that enters self-employment.

Second, while more self-employed co-ethnics provide a larger source of business-relevant knowledge, they also cause more local competition. The greater competition following from more local self-employed co-ethnics could decrease incentives for immigrants to enter into self-employment, particularly if co-ethnics engage in similar business sectors, as previous research suggests (Kerr and Mandorff 2016). Given the simultaneous mechanisms through which ethnic enclaves are related to all sorts of employment, the average effects of both self-employed co-ethnics (resources) and number of co-ethnics (quantity) on immigrants’ entry into self-employment must be decided through empirical investigations.^7^

## 3. The Swedish Case: Immigration, Self-employment, and the Refugee Placement Policy

Immigration to Sweden has increased in numbers and changed in composition over the last half century.^8^ In 1950, less than three percent of the Swedish population was born outside the country, and most originated in other Nordic countries. By the end of 2017, 1.8 million foreign-born individuals composed around 18 percent of the Swedish population, with more than half born outside Europe. This shift in composition should primarily be seen as a change from labor-induced migration from Western European and Nordic countries in the 1950s and the 1960s to refugee and family-related migration from outside the European Union since the 1980s (Dahlberg, Edmark, and Lundqvist 2012).

Regarding self-employment among migrant groups in Sweden, previous research (e.g., Hammarstedt 2004; Hammarstedt and Shukur 2009; Aldén and Hammarstedt 2016; Klinthäll et al. 2016) has suggested a number of general observations, a few of which are particularly relevant to this article. First, many immigrant groups in Sweden have higher self-employment rates than do native-born residents, but those rates vary greatly by source countries in both levels and change over time (Hammarstedt 2004). Second, there seem to be large differences between immigrant men and women, with immigrant men tending to become self-employed to a larger degree than immigrant women (Klinthäll et al. 2016). Third and finally, there is an increasing amount of literature documenting discrimination against self-employed immigrants, potentially from both consumers and financial lenders (Aldén and Hammarstedt 2016). I attempt to address this latter point when presenting the results of this study by regressing self-employment among refugees on capital income within the ethnic enclave (see Table 3).

**Table 1.** Distribution of Country of Birth for Final Sample of Immigrants Who Arrived in 1990 and 1991.

	Freq	Percentage	Cum.
Iran	3,317	22.58	22.58
Iraq	2,098	14.28	36.87
Lebanon	1,980	13.48	50.35
Ethiopia	1,447	9.85	60.20
Somalia	1,395	9.50	69.70
Syria	1,222	8.32	78.02
Yugoslavia	1,039	7.07	85.09
Vietnam	945	6.43	91.52
Romania	717	4.88	96.41
Bulgaria	528	3.59	100.00
Total	14,688	100.00	

**Table 2.** Baseline Estimations (c.f. Equation 1).

VARIABLES	(1) S-E or Not	(2) S-E or Not	(3) S-E or Not
	MUNICIPALITY		PARISH
# Self-employed co-ethnics (As share of municipality pop)	0.0252*** (0.00518)	0.0215*** (0.00555)	0.0154*** (0.00486)
# Non-self-employed co-ethnics (As share of municipality pop)	-0.0162*** (0.00415)	-0.0121*** (0.00450)	-0.00677 (0.00447)
Observations	14,000	14,000	14,000
Mean dep. variable	0.044	0.044	0.044
Covariates and fixed effects	NO	YES	YES

*Notes:* Linear estimations regressing probability of self-employment within five years since residence permit on the standardized share of co-ethnics with business income and municipality population share of all other co-ethnics in 1990 and 1991. ***p < 0.01, **p < 0.05, *p < 0.1. Standard errors (within parentheses) are clustered on municipality level. Column (1) includes no covariates nor fixed effects and column (2) controls for country of birth by cohort, municipality of arrival, age, age2, university education (t+2), sex, marital status, if the individual has children, number of children and if the individual moved within the first year of arrival or not (see equation 1). In column (3) I perform the same estimations as in column (2), only I use parishes instead of municipalities to measure the enclave variables.

**Table 3.** Redoing the Baseline Regressions (Table 2) Using Different Definitions of the Ethnic Enclaves.

VARIABLES	(1) S-E or Not	(2) S-E or Not	(3) S-E or Not	(4) S-E or Not
#Unemployed co-ethnics (As share of co-ethnic mun. pop.)	-0.00305 (0.00311)			
# Co-ethnics w. Capital Inc. (As share of mun. pop.)		-0.000749 (0.00384)		
# High educated co-ethnics (As share of mun. pop.)			0.00835*** (0.00311)	-0.000352 (0.00426)
# Self-employed co-ethnics (As share of mun. pop.)				0.0130*** (0.00472)
Observations	14,000	7,095	14,000	14,000
Mean dep. variable	0.044	0.044	0.044	0.044
Covariates and fixed effects	YES	YES	YES	YES

*Notes:* Definitions of enclaves are: Column (1), unemployed co-ethnics as share of co-ethnics in municipality. Column (2), co-ethnics with capital income, and column (3) co-ethnics with a university education. Note that for column (2), only cohort 1991 is used, since capital income is not seen on individual level in the data in 1990. For more information on specification see Table 2.

One particularly notable component of migration to Sweden has been the arrival of refugees, which in turn has spawned varying settlement polices over time. One such settlement policy was in place between 1985 and 1994 and was aimed at geographical dispersion of refugees.^9^ In brief, the policy implied the following structure: After arrival and application, asylum-seekers in Sweden were placed by governmental agencies in centers distributed all over the country. According to Edin, Fredriksson, and Åslund (2003), there was no correlation between a center’s location and an asylum-seeker’s port of entry. Conditional on receiving a residence permit (at which point the asylum-seeker becomes a recognized refugee), the refugee was placed in one of the contracted municipalities, which, during this article’s time span, included almost all Swedish municipalities (277/284 in 1989; Åslund and Rooth 2007). The municipality received state contributions to finance the reception of arriving refugees; however, refugees were allowed to move after placement, and welfare contributions were not contingent on their staying in the assigned location.

The refugee placement program’s primary goal was to limit refugee inflow to big city regions in Sweden. As has been shown (see Figure 3B in Dahlberg, Edmark, and Lundqvist 2012), it largely succeeded regarding this ambition. Given that the policy aimed to strategically place refugees in a certain manner, it cannot be seen as a randomized experiment. Nonetheless, Edin, Fredriksson, and Åslund (2003) argue that the program can be seen as exogenous from the viewpoint of arriving individuals for a few reasons. First, while refugees were allowed to state preferences on where they wanted to settle, previous research indicates that these suggestions were generally given little consideration (Read 1992; Borevi and Myrberg 2010). Second, as argued by Edin, Fredriksson, and Åslund (2003), since there was no contact between municipal officers and refugees, selection on unobservables is likely ruled out. To the extent that placement was strategic, it was most likely based on information available in the Swedish data registers. We can, therefore, treat the placement policy as exogenous, *conditional on observable characteristics*. This latter point is important, since it indicates that standard balancing tests on observables will not be informative.^10^

While the refugee placement program brings a methodological advantage, questions may remain concerning the relevance of a sample from 1990 and 1991 to current-day refugee dynamics. I claim there are at least two reasons this focus still carries relevance. First, while my primary outcome centers on entry into self-employment within the first five years after arrival, it is also of value to consider ethnic enclaves’ long-term economic outcomes, as I do in section 5.3. For such long-term outcomes, it is necessary to consider refugees who arrived a long time ago, which makes a sample from 1990 and 1991 a highly relevant group. Second, research on Sweden (Ruist 2018) has shown that refugees’ labor-market integration patterns, by source country, have been fairly stable since the 1990s. In other words, the labor-market dynamics for refugees sorted by birth country may not have changed that much over the past decades (Ruist 2018).^11^ Summing up, then, there are sound theoretical and empirical reasons for using data from the years 1990 and 1991. The details of the data, as well as the empirical methods used to analyze it, are the topics of the next section.

## 4. Data and Empirical Model

To estimate ethnic enclaves’ effect on the probability among refugees to become self-employed, I make use of GeoSweden, a large and rich administrative database with yearly, individual information on all residents in Sweden from 1990 to 2014. The information is collected by Statistics Sweden and includes, among other things, demographic and socio-economic information, as well as source country and the date when an immigrant (including refugees) obtained his/her residence permit. The latter information is important to notice: Data are available for all immigrants *after* they obtain residence permits, not before. Place of stay, which is used for inference on refugees’ assigned municipality, is registered on December 31 of every year.

The sample consists of working-age (18–55 years old), foreign-born adults who arrived and were given a residence permit in Sweden during 1990 or 1991. The choice of years is related to the identification strategy, which makes use of the already-described refugee placement policy. The policy was in place between 1985 and 1994, but reportedly became less encompassing after the unexpected increase in immigration from former Yugoslavia in 1992 (Åslund and Rooth 2007). Given that the database does not stretch further back than 1990, the first two years of the 1990s compose the sample.

While the database has several unique features, there are limitations. One clear limitation is the lack of information on the reason for immigration. This limitation is at the heart of the research design, since only refugees were placed through the settlement program. To make sure my sample is first and foremost composed of refugees, I add two restrictions. First, I limit the sample to those who were born in a non-OECD country. Of this group, I keep only the 10 birth countries with the largest immigration to Sweden in 1990 and 1991. The Swedish Migration Agency documented high levels of refugee intake from these countries, but no or very little work or student migration.^12^ Second, I drop all individuals who already had a household member living in Sweden in a prior year. This criterion is crucial, since arriving migrants with family members in Sweden could have applied as tied family migrants, implying they were not subject to placement via the governmental program. The two restrictions on having a specific birth country and no family already settled in Sweden leave me with a sample that most likely includes very few non-refugees. Note that for simplicity, I refer to all individuals in my sample as refugees.

Some refugees, after being settled in a municipality, moved to another municipality before the end of the year of arrival. Since the data only allow me to observe the place of residence on December 31, it follows that for some refugees, the municipality of residence observed in the data will be different from the one in which refugees were initially placed. I do, however, have access to a binary variable stating if individuals moved between municipalities during a year. To make sure the choice of moving is not systematically related to the enclave size and the choice of self-employment, all regressions include moving or not moving during the first year as a control variable. The total sample includes 14,688 individuals from the 10 countries shown in Table 1.^13^

A few characteristics about the refugees in the sample deserve mentioning. First, an average refugee in the sample came to a municipality with 403 adult co-ethnics, of whom on average 2.5 percent, or 16 individuals, were self-employed. Second, around 4.5 percent of the sample became self-employed at any point during the first five years in Sweden. Of these, a fairly large portion started restaurants, the business sector for almost 40 percent of self-employed refugees studied here. Other important sectors included retail stores, hairdressers, and cab drivers. Third, with regard to individual characteristics, self-employed refugees were similar to the sample at large in terms of education level, parental status, and the likelihood of being placed outside big cities. What is strikingly different between self-employed refugees and the rest of the sample, however, is that an overwhelming share (>80 percent) of the businesses were run by men. The large male overrepresentation among the self-employed suggests that the results in this article are valid first and foremost for men. At the very least, the overrepresentation of male over female self-employed refugees motivates heterogeneity analysis by sex. Results from the heterogeneity analysis confirm that my conclusions hold primarily for male refugees (see Section 5.2). More detailed descriptive statistics on the sample of refugees can be found in the online Appendix, Section A, Tables A1 and A2.

### 4.1 Empirical Model

Given the sample selected, the goal is to estimate the causal effect of different measures of the ethnic enclave on the probability of self-employment. Entry into self-employment is a binary variable, which takes the value 1 if an individual declares positive business income and 0 otherwise.^14^ This definition of self-employment is broad for two reasons. First, I wish to maximize the number of observed self-employed. Second, since I am interested in *entry into* self-employment, the size of business income or hours worked are not primary issues. To make sure my results are not dependent on the definition of self-employment, I do, however, provide robustness results in the online Appendix (see Table B1), using alternative definitions of the dependent variable, and my main findings are not sensitive to the definition of self-employment.

In the baseline estimate, the dependent variable is measured within five years and is cross-sectional in nature. Starting a business may take time, so I do not want a time horizon that is too short; on the other hand, if the lag is too long, the connection to the network in the assigned municipality likely becomes less important. I, thus, chose five years as a midway case.^15^

My research design implies that, for individual *i*, born in country *c*, arriving in municipality *m*, with cohort *k* (1990 and 1991), I regress whether the individual earned business income at some point within five years after arrival, on the share of self-employed co-ethnics and share of other co-ethnics in the municipality of arrival.^16^ Fixed effects are included for arrival municipality ( $\theta_{\text{m}}$ ) and the interaction of cohort and birth country ( ${\text{δ}}_{\text{kc}}$ ). I further add a vector of individual-level covariates ( $X_{\text{it}}$ ), including age, ${\text{age}}^{2}$ , sex, university degree, if the individual moved during the arrival year, if he/she was married, if the individual had children, and how many. The full specification is seen in equation 1 (where **SE** = self-employed and **nonSE** = not self-employed).


1 $$y_{icmk}=\beta_{0}+\beta_{1}\text{*}\frac{\#\mathrm{SE}\;coethnics_{mck}}{Population_{mk}}+\beta_{2}\text{*}\frac{\#non\mathrm{SE}\;coethnics_{mck}}{Population_{mk}}+X_{\text{it}}+\;\theta_{\text{m}}+\;\delta_{\text{kc}}+{∊}_{icmk}$$

#### Identification discussion

This article seeks to identify the effect of co-ethnics on refugees’ behavior (in this case, the decision to start a business), but as formalized by Manski (1993, 2000), the fact that an individual behaves similarly as a chosen peer group could be due to correlated attributes within the group. Also, if the group affects the individual’s actions, the reverse could be true. In the current case, for identification of the relevant parameters $\beta_{1}$ and $\beta_{2}$ , I require the arrival-year characteristics of the location, conditional on individual covariates and fixed effects, to be independent of attributes related to entry into business and the share of co-ethnics/share of self-employed co-ethnics.

The design in Equation 1 deals with most of the correlated characteristics, which may affect the outcome of refugees and their peers. Local labor-market effects and any general tendency among refugees from a certain country and cohort to become self-employed are controlled for with fixed effects for municipality and country by cohort. The fixed effects, however, do not address possible selection into municipalities. To account for selection, I use the logic of the dispersal policy, which, conditional on a number of observed individual characteristics, stripped away the possibility to choose one’s place of stay.

While there is no definitive way to test the assumptions underlying the empirical model, I provide a few additional results supporting my research design. Most importantly, I want to make sure that individuals with a higher ex-ante probability to start a business did not for different reasons end up in larger enclaves. I, therefore, use a linear regression model to predict the probability of self-employment as a function of a battery of individual characteristics, all related to entry into self-employment.^17^ I then regress the municipality share of self-employed co-ethnics or of all co-ethnics *on* predicted self-employment. Arguably, there should be no effect on enclave size from a higher probability of self-employment. The coefficient is negative, non-significant, and as low as 0.001, which arguably is very low. Note also that since the effect is negative, if there is any selection of those more prone to self-employment, they seem to choose municipalities with fewer co-ethnics. Arguably, it is less of a problem if refugees who are more prone to self-employment end up in smaller, rather than larger, enclaves, since the former story would imply an underestimation of the enclave effect on refugee entry into self-employment with my research design.

Lastly, one could also imagine unobserved labor-market characteristics at the municipality level, especially fitting for refugees from a certain birth country. Such labor-market characteristics could drive both the self-employment tendency for newly arrived refugees and the number of co-ethnics who came to the municipality in previous years. I, therefore, include robustness tests where I add controls on municipality employment rate among co-ethnics, which captures a specific country group’s municipality labor-market integration. Indirectly, a control for municipality employment rate among co-ethnics further tests whether individuals become self-employed due to poor labor-market integration in a certain municipality. My robustness checks suggest that this mechanism is not the driving force behind the results observed (see the online Appendix, Table B2).^18^

## 5. Results

In Table 2, I report results from the estimation of equation 1, with the main treatment variables standardized (mean = 0, standard deviation = 1). The coefficients hence represent the effect of a standard deviation increase in the explanatory variable. Column (1) is a linear regression excluding all covariates and fixed effects, while column (2) adds individual controls and dummies for municipality of arrival and birth country by cohort. Standard errors (within parantheses) are clustered at the municipality level.

A first striking feature is that the estimations in columns (1) and (2) are fairly stable with regard to the effect of self-employed co-ethnics. Adding covariates and dummies changes the average effect very little, which is reassuring in terms of exogeneity. The effects are statistically significant at the 1-percent level when fixed effects are included. In the preferred specification, a standard deviation increase in the share of self-employed co-ethnics gives a two percentage point increase in self-employment propensity. Given that only 4.4 percent of those who received residence permits in 1990–1991 had business income at some point within five years, the estimated effect is large (45 percent of the base-point). It is, however, important to keep in mind that around 25 percent of refugees were placed with self-employed co-ethnics, while only around 15 percent were placed with more than one standard deviation. A reasonable way to look at the treatment effect is, therefore, as an increase from a municipality with no or very little presence of self-employed co-ethnics to a municipality with a large presence of co-ethnics with a business. The coefficient hence reflects a large effect stemming from a fairly large treatment.

While the estimates using the share of self-employed co-ethnics are both sizeable and significant, the coefficient representing the quantity of all other co-ethnics is actually negative. The negative coefficient implies that, given a certain share of self-employed co-ethnics, a larger share of other co-ethnics actually decreases the probability of entry into self-employment among refugees. A standard deviation increase in the share of co-ethnics gives a one percentage point drop in the probability of self-employment. There are several possible interpretations of this coefficient, but, most importantly, the overall number of co-ethnics does not seem to play a big role in shaping the connection between ethnic enclaves and entry into self-employment among refugees in Sweden.^19^

A key choice in the model estimated in Table 2, columns (1) and (2), is that the ethnic enclave is defined using the share of co-ethnics or self-employed co-ethnics in the *municipality*. The primary reason for using municipalities as a geographical context is that there needs to be a substantial amount of co-ethnics for me to credibly claim the existence of a co-ethnic market. The number of municipalities used in the estimations is, however, only 188, and one might worry that there is not enough variation to separate the effect of self-employed co-ethnics from that of co-ethnics in general. For this reason, I also provide the same estimations, but for a lower geographical level — parishes. Parishes are smaller in size: the average parish population in the sample is only 8,500, and refugees are spread over 550 parishes. The enclave size in a parish is naturally also smaller, with on average 75 co-ethnics, of which three were self-employed.

The coefficients for the parish estimations are shown in column (3). Again, the share of self-employed co-ethnics increases the probability of self-employment, while no such effect can be detected for the share of all co-ethnics. The latter coefficient is still negative, but the size is very small and the estimate is not significantly different from zero.

Besides the level of geography, the estimates could be sensitive to many things. In a series of robustness checks, I, therefore, re-estimate the baseline case with different techniques and samples. First, I include a specification with a different definition of the dependent variable, which is based on self-employment as the main income source (Table B1). Furthermore, I test for the inclusion of additional control variables, including a quality indicator for birth country at the municipality of arrival: the local employment rate within an ethnic group (Table B2). I also include estimations using non-linear specifications (probit and logit, Table B5) and different functional forms, including the absolute number, log, and an inverse hyperbolic sine transformation of co-ethnics (Table B6). The overall conclusion from the sensitivity checks is that the effect of being placed with self-employed co-ethnics remains both positive and significant, while the effect of *all* co-ethnics stays significant and negative, or insignificant. The sensitivity checks are all found in the online Appendix, in Section B.

### 5.1 Investigating the Channels Further

A key alternative story to the importance of co-ethnic networks is that refugees could become self-employed because they lack skills required in the formal labor market or because they encounter discrimination (Aldén and Hammarstedt 2016). If this story is valid as an explanation in the current setting, there must be discrimination specific to a country *and* a municipality. Under this arrangement, a larger share of self-employed co-ethnics could reflect difficulties on the local labor market. I attempt to test for the discrimination/lack of skills story more extensively by looking at the unemployment rate of resident co-ethnics at arrival. I divide the number of unemployed co-ethnics by all co-ethnics within a certain municipality, which gives me a measure for how poorly a group of co-ethnics is doing or how discriminated they are in their municipality of residence. Results using the unemployed co-ethnics can be found in column (1) of Table 3. The coefficient implies that a standard deviation increase in the share of unemployed co-ethnics decreases the probability of self-employment by 0.003 percentage points. It, therefore, seems unlikely that the story of a lack of formal requirements and discrimination is driving the baseline results.^20^

Another explanation for the results is access to financial assets. This explanation, however, does not fit with the results observed. In column (2) of Table 3, I regress the dependent variable on the share of the municipality population who were co-ethnics with capital income. The coefficient is insignificant, small, and negative. Capital income among co-ethnics is, therefore, not a strong predictor of self-employment.^21^

Lastly, other resources among co-ethnics could matter. I, therefore, define a set of high-resource individuals, counting co-ethnics with a university education. As in the baseline case, I also standardize the co-ethnics with municipality population. As can be seen from the estimates in column (3) of Table 3, there is indeed a significant effect from living close to those with a university education. The effect is smaller than the baseline estimate of self-employed co-ethnics, but still economically significant. However, when adding a control for the standardized share of self-employed co-ethnics, the effect disappears (c.f. column 4). A word of caution is that all the definitions used in Table 3 are highly correlated.

### 5.2 Extension 1: Heterogeneity

As an additional exercise, I redid the baseline analysis (Table 2, column 2) separately for eight subgroups, which have all been linked to a higher (or lower) degree of self-employment (Simoes, Crespo, and Moreira 2016). I separated the sample into high- and low-educated (university educated and those with less than nine years of education), men and women, married and non-married, and parents and non-parents. The results can be seen in Table 4.

**Table 4.** Heterogeneity Estimations.

VARIABLES	(1) University Educ. (t+5)	(2) *<* 9 Years of Educ.	(3) Men	(4) Women	(5) Married	(6) Non-married	(7) Parents	(8) Non-parents
# Self-employed co-ethnics (as share of municipality pop.)	0.0247** (0.0114)	0.0233*** (0.00727)	0.0383*** (0.00947)	0.00157 (0.00328)	0.0134*** (0.00452)	0.0277*** (0.00910)	0.0173** (0.00752)	0.0219*** (0.00631)
# Non-self-employed co-ethnics (as share of municipality pop.)	-0.0221* (0.0118)	-0.00820 (0.00606)	-0.0197*** (0.00655)	-8.07e-05 (0.00348)	-0.00456 (0.00451)	-0.0191*** (0.00749)	-0.00805 (0.00686)	-0.0137** (0.00529)
Observations	3,020	6,601	8,759	5,241	7,467	6,533	5,042	8,958
Covariates and fixed effects	YES	YES	YES	YES	YES	YES	YES	YES

*Notes:* Heterogeneity results using subgroups of the cohorts.

A few interesting conclusions can be made from the heterogeneity results. First, while there are some differences in the estimated coefficients depending on civil status and parenthood, the conclusions are qualitatively alike. Similarly, there is no distinguishable difference between educational groups. Second, the coefficient for women is insignificant and small, suggesting no effect of ethnic enclaves on female entry into self-employment. That the effect is driven by males is in line with previous findings showing that more businesses are opened by men (Simoes, Crespo, and Moreira 2016). Suggested explanations for the differences between men and women include discrimination from lenders, men and women’s involvement in different business sectors, and women’s tendency to be more risk averse (Simoes, Crespo, and Moreira 2016). The small share of women in my sample who opened a business makes it hard to develop the analysis further (more than 80 percent of businesses in the sample were registered to males). Thus, an interesting task for future research is to consider how female and male networks may differ and why.

### 5.3 Extension 2: How Do the Self-employed Perform?

As a last extension, I turn to the long-term economic outcomes. My aim with this section is partly to study whether the residential concentration of self-employed co-ethnics has a long-term economic impact and partly to determine whether the effects, if they exist, are counteracted or enhanced by the choice of early entry into self-employment. I address this complex issue using mediation analysis.^22^

I consider two outcomes over a 20-year period: disposable income and social assistance.^23^ Income is to some degree the intuitive outcome to study, but income from self-employment is plagued with uncertainty. Business income is evidently easier to hide or mask than other labor income, since the latter is generally reported by a third party. Potential underreporting of business income can partly be dealt with by scaling the portion of disposable income registered as self-employment income upwards (with 1.6 in accordance with methods used by Statistics Sweden), but for a more complete picture, I also include the measure of social assistance.

Essentially, the mediation analysis is done in two steps (I use the case of social assistance to illustrate the method). First, I fit the baseline model (equation 1), estimating the effect of share of self-employed co-ethnics on entry into self-employment $\left(\beta_{1}\right)$ . Second, I regress the aggregated social assistance on the same right-hand-side variables as in Equation 1; only I now add a dummy representing if the individual entered self-employment during the first five years as a right-hand-side variable (i.e., the dependent variable in equation 1). In the second case, I estimate the effect of entry into self-employment on long-term social assistance, *conditional on ethnic enclave size and pre-treatment characteristics*. The product of (a) the effect of self-employment on social assistance and (b) the effect of ethnic enclaves on self-employment entry gives the mediation effect. In other words, assuming there is an effect of ethnic enclaves on long-term social assistance among refugees, how much is mediated through the choice of self-employment?^24^

Table 5 shows the average direct effect and the percentage of the total effect mediated by self-employment. As can be seen, being placed in a municipality with a higher share of self-employed co-ethnics has a negative effect on aggregate disposable income. The estimate suggests that a standard deviation increase in the share of self-employed co-ethnics makes long-term income drop by 5 percent. Table 5 also shows that the choice of self-employment is associated with a slightly lower income, making up around 1 percent of the total enclave effect. There is, hence, a small economic penalty connected to ending up in a municipality with more self-employed co-ethnics, and if we believe the mediation estimates, this negative relation is not affected by entry into self-employment to a very strong degree. In column 2, the enclave’s negative influence is again seen, with the sum of social assistance increasing as a function of the share of self-employed co-ethnics. In this case, the effect is seen in absolute numbers, given in hundreds of SEK.^25^ A coefficient of 41, hence, implies an increase of 4100 SEK over the next 20 years, which represents less than 2 percent of the mean level of social assistance for this sample. In the case of social assistance, we do, however, see a counteracting mediation effect of entry into self-employment, making up more than 30 percent of the total effect.

**Table 5.** Causal Mediation Effect of Self-employment on Aggregate Disposable Income over 20 years (95 Percent Confidence Intervals in Brackets).

AVERAGE EFFECTS	(1) ln(Disposable Income)	(2) Social Assistance
Causal mediation effect	0.0005	-20.48357
	[-0.0011, 0.0020]	[-28.5965, -12.7045]
Direct effect	-0.0510	62.2575
	[-0.0781, -0.0222]	[-14.7643, 144.4315]
Total effect	-0.0506	41.7739
	[-0.0771, -0.0217]	[-35.1142, 125.4353]
% of tot effect mediated through S-E	-0.009	-0.333
	[-0.0217, -0.0061]	[-5.8496, 5.2665]

*Notes:* Causal mediation analysis following the procedure in set out in Hicks and Tingley (2011).

“Total effect” represents the effect of the size of the ethnic enclave on aggregate log disposable income in (1) and social assistance in (2). % of tot effect mediated through S-E is the share of the total effect given by the mediator (self-employment). In column (1), the top 1 percent of income earners are dropped, and the share of income that stems from self-employment is multiplied by 1.6. Social Assistance is measured in hundreds of SEK. The mean aggregate social assistance is 240,000 SEK.

All in all, then, the results show that self-employment entry has a close to zero mediation impact on disposable income but lowers the negative impact of the ethnic enclave measure on aggregate social assistance. The results, hence, suggest a possible positive influence of early entry into self-employment, in that it significantly decreases the negative relation between share of self-employed co-ethnics in the municipality of arrival and social assistance received by refugees the following 20-year period.

How sensitive are the mediation results to possible endogeneity? Imai, Keele, and Tingley (2010) have developed a sensitivity check to further analyze the importance of unobserved variation, in this case affecting both the long-term outcomes and entry into self-employment. The method relies on simulating the average mediator effect depending on different assumptions about the unobserved variation. More exactly, I plot the simulated average mediation effect, given different values of the correlation between the residuals of equation 1 and of an equation with the long-term outcome as dependent variable.^26^ Focusing on the outcome of social assistance, a negative correlation between the residuals implies that individuals with a lower social assistance than predicted (high-ability individuals) become self-employed (more than predicted).

**Figure 1.**
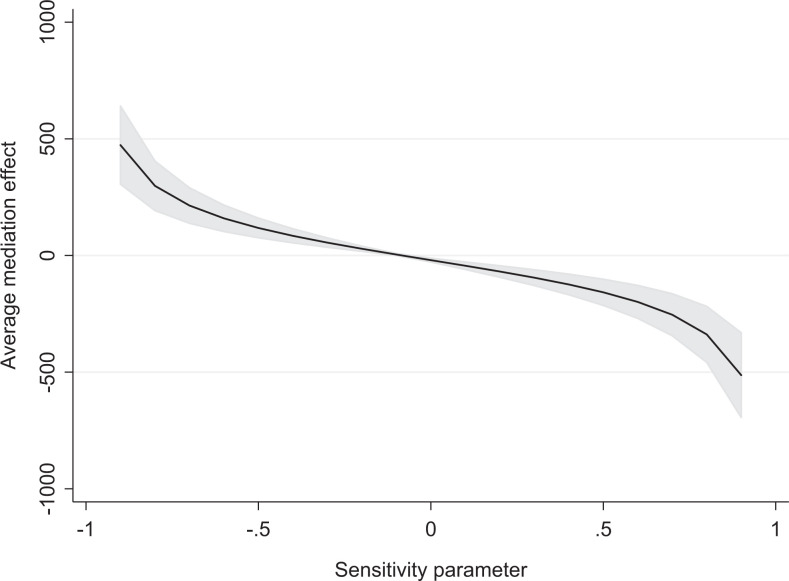
Sensitivity Check of Mediation Analysis.

The results are seen in Figure 1, which shows that a negative correlation of around -0.08 flips the sign of the simulated average estimate. In essence, if more marginalized, less able individuals become self-employed (more than expected), the mediation estimates in Table 5 (which suggested self-employment could counteract the negative relation between the share of self-employed co-ethnics in the municipality of arrival and the use of social assistance) may even be an underestimation. If, however, individuals selecting into self-employment are more able than expected from individual characteristics, the effect on both income and social assistance may hide a much more negative picture.

## 6. Discussion

This article utilized a spatial dispersal policy in place from 1985 to 1994 in Sweden to estimate ethnic enclaves’ causal effect on entry into self-employment. The results indicate that refugees who were settled in municipalities with a higher share of *self-employed* co-ethnics also had a higher probability of entering self-employment in the coming years. This main result was robust to several different specifications and functional forms. On the contrary, being placed in a municipality with a large number of co-ethnics, regardless of characteristics, had no, or even negative, effects on the probability of self-employment. Moreover, neither being settled with more co-ethnics with capital assets nor being settled with more marginalized/unemployed co-ethnics had any statistical relation to entry into self-employment among refugees.

The estimates are, hence, first and foremost consistent with the story that information sharing and know-how (resources) from co-ethnics already familiar with the market or important institutions cause an increased entry into self-employment (Light, 1984). Whether the information and know-how relate to the legal framework, specific self-employment skills, language, or other institutional knowledge is difficult to pinpoint with the data at hand. Nonetheless, the interpretation that know-how and information are the main drivers of the ethnic enclave stands in contrast to the main alternative story (e.g., Borjas 1986), which promotes the argument that access to a large market of potential co-ethnic consumers (or workers) increases self-employment.

With regards to the empirical literature on the specific relation between ethnic enclaves and self-employment, previous results have been mixed. Like Yuengert (1995), Clark and Drinkwater (2002, 2010), and Damm (2009), but in contrast to Borjas (1986), Le (2000), and Aldén and Hammarstedt (2015), I find no evidence of a positive relation between enclave size and immigrant entry into self-employment. A problem is, however, that almost all these studies consider only enclave size, with little or no focus on *resources within* the enclave. Also, besides Damm (2009), previous research has not addressed the issue of omitted variable bias. Thus, comparisons to associative studies should be done with some care, since they do not fully address issues of endogeneity, which implies that results, to a varying degree, may be biased.

While the share of self-employed co-ethnics at the municipality of arrival affects refugees’ probability of self-employment, on average, the results suggest a small income penalty of being placed with more self-employed co-ethnics. However, using mediation analysis, I show that any long-term negative effects are potentially counteracted by early entry into self-employment. The mediation analysis is, however, sensitive to selection into self-employment. Should it be that more able individuals select into self-employment, the story may be the opposite. Due to the difficulties of overcoming issues of selection into different forms of employment, an interesting venue for future studies is to gain a better understanding of the characteristics of those who select into self-employment.

The main conclusion of this article is that rather than the ethnic enclave’s size per se, the key factor in determining refugee entry into self-employment in Sweden is the *resources within* the enclave, reaffirming a more general result seen in other studies of the economic effects of ethnic enclaves. For example, both Edin, Fredriksson, and Åslund (2003) and Damm (2014) found that different measures of enclave quality, rather than size, cause an increased entry into employment among immigrants. Also, Åslund and Fredriksson (2009) found no relation between enclave size and entry into welfare among refugees in Sweden, whereas the presence of more co-ethnics *on welfare* increased refugees’ probability of collecting social assistance. These results, as well as the results of this article, call for moving away from using only ethnic enclave size and toward focusing on varying measures of resources, attributes, and qualities within ethnic enclaves.

## Supplemental Material

Supplemental Material, SI_final - Ethnic Enclaves, Self-employment, and the Economic Performance of Refugees: Evidence from a Swedish Dispersal Policy Supplemental Material, SI_final for Ethnic Enclaves, Self-employment, and the Economic Performance of Refugees: Evidence from a Swedish Dispersal Policy by Henrik Andersson in International Migration Review

## Notes

1 Two individuals are defined as “co-ethnics” if they are born in the same country. This definition is due to the database used, which includes information on birth country, but not ethnicity. Birth country is hence used as a best available proxy for ethnicity. I further extend the definition in the results section, approximating ethnicity by language spoken in the birth country.

2 Another closely related study is Andersson, Larsson, and Öner (2017), who examine the association between ethnic enclaves and self-employment among Middle-Eastern immigrants in Sweden, focusing on very small neighborhoods (1 km^2^).

3 As I discuss in more depth when presenting the results, this long-term positive impact of entry into self-employment assumes that very few high-ability refugees select self-employment.

4 See also Kalnins and Chung (2006), who find longer survival rates for Gujarati Indian-owned hotels when more hotels in the vicinity are owned by co-ethnics. For more general reviews on entrepreneurship and ethnicity (or immigration), see Aldrich and Waldinger (1990) and Fairlie and Lofstrom (2015).

5 Treating co-ethnics as peers is common in the empirical literature on ethnic enclaves and economic outcomes, and has been argued to be a natural starting point by previous research (Åslund and Fredriksson 2009). That said, non-coethnics with relevant skills may also affect the probability of self-employment. Much like previous research (e.g., Edin, Fredriksson, and Åslund 2003; Åslund and Fredriksson 2009; Eliasson 2014), I, however, include fixed effects for the geographical unit (municipalities) in my regressions. As a result, I cannot identify the effect of non-coethnics’ skills since this measure is identical for all individuals within a municipality.

6 An important nuance is that while there are comparative advantages in selling products to a specific ethnic group, it can also limit firm growth. See, for example, Aguilera (2009), who finds that self-employed Mexican immigrants residing within enclaves in the United States have lower returns than Mexican immigrants residing outside such enclaves.

7 A last important consideration is that individuals might suffer from liquidity constraints, which can be eased with access to co-ethnics with assets (e.g., Eliasson 2014). To test for the importance of co-ethnics with assets on entry into self-employment in the current setting, I run regressions showing that being placed with more co-ethnics with capital income does not cause a higher probability of self-employment.

8 All numbers on immigration and the foreign-born population presented in this section are found or can be constructed from the website of Statistics Sweden at https://www.scb.se/hitta-statistik/statistik-efter-amne/befolkning/befolkningens-sammansattning/befolkningsstatistik, and the file “Folkmängd efter födelseland, 1900-2018,”.

9 The section on the refugee placement program draws on various sources including Read (1992), Invandrarverket (1997), Edin, Fredriksson, and Åslund (2003); Åslund and Fredriksson (2009), Borevi and Myrberg (2010), and Dahlberg, Edmark, and Lundqvist (2012).

10 Many key variables, however, still balance well. Most importantly, conditional on birth country, university education and enclave size are not correlated, regardless of whether I use education measured in the arrival year or five years later.

11 That said, in the early 1990s, Sweden went through a large financial crisis with severe labor-market effects (Åslund and Rooth 2007). The crisis may have led to unusually long unemployment spells for new immigrants, particularly refugees — a situation that might have increased the relative attractiveness of opening a business for this group. Assuming the latter is true, it should be the case for all refugees. In other words, while one could easily see how the crisis affected the general tendency among refugees to become self-employed, it is less clear that it would change the effects of ethnic enclaves within specific birth countries.

12 See the online Appendix, Section A, for more detailed information.

13 Note that the sample consisted of 14,688 individuals who came in 1990 and 1991, but 600 of them left Sweden between 1990/1991 and 1995/1996. The distribution of birth country for those emigrating fairly well matches those staying.

14 To be exact, only business income that is active, rather than passive, is included. “Active” income is related to tax liability and is, in theory, based on the self-employed having worked at least 600 hours during the relevant year or performed the operation with his/her own effort.

15 I have estimated models with three- to-seven-year spans as well, giving very similar results to the five-year baseline case. These results are available upon request.

16 I use municipalities as my geographical reference because I wish to observe ethnic markets, in which case many Swedish neighborhoods may be too small. That said, the choice is not without issues. Detecting differences between enclave resources and size is harder with larger geographical areas that provide less variation than does the neighborhood level. I therefore also provide estimates using parishes, which are closer to the definition of a neighborhood.

17 The individual characteristics include age, age^2^, sex, marital status, whether the individual had children, how many children, if he/she had a university degree, yearly disposable income in the arrival year, social assistance from the state, whether the individual was employed in the arrival year, whether the individual moved the initial year, a dummy for cohort and birth country, and interactions between age and education status, as well as education status and sex.

18 A possible different issue regarding interpretation of the results has to do with subsequent movers. If entrepreneurs who are placed with many self-employed co-ethnics experience too much competition and move to start a business in another municipality, the effects of self-employed co-ethnics at the arriving municipality may reflect the effect of competition rather than the effect of peers. This story, however, does not fit with the data. Enclave size is bigger in 1990 and 1991 for those who stayed put, compared to movers. This difference lessens over the next five years, as subsequent movers seem to be attracted to enclaves, rather than moving away from them.

19 There are at least two objections to this conclusion. First, it could be that a niche-market business is profitable to start only when none already exists. Therefore, in Table B3, column (1), I interact the number of self-employed co-ethnics at arrival with all other co-ethnics and show that there is no significant effect from the number of non-self-employed co-ethnics when the number of self-employed co-ethnics is zero. Second, the ethnic enclaves might be too narrow. A niche market might, for example, have an Arabic, rather than a specific Iraqi, base. In columns (2) and (3) of Table B3, I switch the definition of an ethnic group and focus on language groups instead. The Swedish register data hold no information on spoken language, meaning that any information must be inferred from the most commonly spoken languages in the origin country. Looking at the signs and sizes of the estimates in Table B3, there is no detectable positive effect on self-employment from a larger enclave based on language. The full definition of the language enclaves is found in Table B4. Both Table B3 and B4 are available in the online Appendix.

20 Furthermore, in Table 4, I redo the baseline regressions for different subgroups of the sample, most importantly including having a university degree or not. The estimates are based on education in 1995–1996 and they show no statistical difference in effects between different education levels. Hence, it does not seem that refugees with low education levels, who potentially lack relevant labor-market skills, are reacting more to the effect of enclaves.

21 I also run the model using the share with high capital income (top quartile of capital income earners in Sweden, within a specific birth country). Results available upon request.

22 The mediation approach based on the potential outcome framework is discussed in Imai, Keele, and Tingley (2010). I run the model using the algorithm developed by Hicks and Tingley (2011).

23 Social assistance is welfare support given to those who lack sufficient means to support themselves. Disposable income is a compound measure of all net income, including different types of benefits. Both variables are based on household information, calculated as the individual share of the household income/social assistance. Note, however, that while the individual aggregation is made over 20 years, I can only observe both measures until an individual reaches the age of 64. The latter limitation, combined with the fact that refugees may exit the sample due to emigration or death, means that for some individuals, the number of years counted may be significantly less than 20. As a simple exercise to consider this issue, I regress the binary variable “exit or not” (1 if you exit the data before 2014) on the two measures of the ethnic enclave. The key one, share of self-employed, shows no significant relationship with exit rates. While I cannot fully exclude the possibility that there is a relation between unobserved predictors of income, ethnic-enclave size, and exit tendency, this exercise at least gives suggestive evidence of nothing major affecting the interpretation of results.

24 An important limitation of the research design is that in addition to the already-discussed exogenity assumption (see Section 4.1), an additional assumption is needed for a *causal* mediation interpretation. Given pre-treatment characteristics and enclave size, the choice of self-employment should be independent of the potential outcome: aggregated income or social assistance over 20 years. This assumption is naturally a very strong one. Should there be any unobservable characteristics driving both income or social transfers and the choice of self-employment, estimates will be biased. The direction of the bias depends on what we choose to assume about the unobserved characteristics among those who select into self-employment, and exactly what to expect here is not obvious. As an example, an analysis of Swedish male wage-earners showed that both individuals who earned below predicted income (low-ability individuals) and wage earners earning above predicted income (high-ability individuals) selected into self-employment (Andersson Joona and Waldensjö 2013). I provide sensitivity checks to shed further light on potential problems with endogeneity (see Figure 1).

25 Swedish currency. In 1990, 1 dollar≈6 SEK.

26 Essentially: ${\text{Social Assistance}}_{icmk}=\gamma_{0}+\gamma_{1}\text{*}\frac{\#\text{SE }coethnics_{mck}}{Population_{mk}}+\gamma_{2}\text{*}\frac{\#non\text{SE }coethnics_{mck}}{Population_{mk}}+\gamma_{3}\times y_{\text{icmk}}+{\text{X}}_{\mathrm{it}}+\;\theta_{\text{m}}+\;\delta_{\text{kc}}+{∊}_{icmk}$ .
