# Supplementary material for: Ethnic Enclaves, Self-employment, and the Economic Performance of Refugees: Evidence from a Swedish Dispersal Policy
Source: Int Migr Rev. 2020 May 5;55(1):58–83. doi: 10.1177/0197918320912195 (PMC13029397; doi:10.1177/0197918320912195)
Supplement: Supplemental Material, SI_final - Ethnic Enclaves, Self-employment, and the Economic Performance of Refugees: Evidence from a Swedish Dispersal Policy [file SI_final.pdf]

Supplementary information to *Ethnic Enclaves, Self-Employment and the Economic Performance of Refugees – Evidence from a Swedish Dispersal Policy*

Henrik Andersson

A The sample

Only refugees were placed through the refugee placement program, but for individuals who arrived prior to 1997, the reason for immigration (type of residence permit) is not available in the data. Due to these limitations, I take several steps (described in Section 3) to drop non-refugees. First, as a validation check, I further compared my countries (seen in Table 1) to aggregate historical data available on the Swedish Migration Agency's website.[1] This comparison is not perfect: the GeoSweden data are based on birth country, while the Migration Agency data are based on citizenship. However, as a rough comparison, it is good to see that the numbers do not differ too much. In fact, of the top ten countries for asylum in Sweden in 1990 and 1991, 9 are also in my sample. The only exception is Turkey, which was excluded from my sample on the basis of being an OECD country. One can further note that there were almost no guest students among the countries

[1] See https://www.migrationsverket.se/English/About-the-Migration-Agency/Facts-and-statistics-/Statistics/Overview-and-time-series.html, retrieved 2019-11-20.

in the sample. Also, Migration Agency statistics show that very few individuals from the ten countries used in my sample received labor-market permits. Labor-market permits issued in 1990 and 1991 are not published but were sent to the author from the Migration Agency and are available upon request.

A.1 Some summary stats

Table A1 includes a left panel with individual information for all refugees at arrival (placement year) and the same follow-up information five years later. At arrival, around half the sample was married, and there were more men than women. Furthermore, only around six percent of the sample was highly educated within two years after arrival, while a large majority had less than a high-school education. Notice that, to account for the poor quality of the information on immigrants' education in the year of arrival, I use the reported level of schooling two years after arrival. The education variable is taken from the Swedish education registers, which in this case is divided into seven steps, with 5-7 representing any education above 12 years (gymnasium). I label schooling above 12 years as high education. Low education includes those with 9 years or less, or with no information on education.

Looking at the key variables, *#coethnics* means that an average refugee in the sample came to a municipality with 403 adult coethnics, of whom 200 had a positive salary (# *coethnics with wage>0*) and almost 16 were self-employed (# *S-E coethnics*). Seen as share of the number of coethnics, on average about 2.5 percent of coethnics were self-employed, and seen as share of the full population, around 0.02 percent were self-employed coethnics. Lastly, and not surprisingly,

Share with Business Income says that only ten arriving migrants were able to start a business within their first year in Sweden. Five years later, around 3 percent of the sample had some business income. It is important to remember that 3 percent reflects the share of the entire sample, in which more than half were unemployed. Seen as a share of the employed, the rate of self-employment was almost 12 percent.

In Table A2, I continue by showing characteristics and type of establishment among those who became self-employed. 611 individuals had some business income within the five-year interval, representing around 4.5 percent of the sample. The share of high and low educated seem to be the same as the sample at large, which also goes for the share of parents and the share placed outside big cities. The self-employed were slightly younger than the rest of the sample, but most importantly, the share of men was overwhelming. Over 80 percent of the establishments were run by men. Unfortunately, a sizable portion of individuals owning firms had missing values on sector information. Of those left, most worked in five sectors seen in the upper panel of Table A2.

Table A1: Summary Statistics

VARIABLE	1990-1991 Arrival year Statistics			1995-1996		
Individual characteristics	N	Mean	Std.dev	N	Mean	Std.dev
Age	14,668	30.66	8.48	14,000	35.58	8.43
Married	14,688	0.53	0.50	14,000	0.62	0.49
Men	14,668	0.62	0.48	14,000	0.63	0.48
Children	14,688	0.36	0.48	14,000	0.54	0.50
#Children (| parent)	5,249	2.22	1.27	7,610	2.28	1.31
Highly educated (t+2)	14,688	0.06	0.24	14,000	0.22	0.41
$\leq$ 9 years of education (t+2)	14,688	0.79	0.41	14,000	0.47	0.50
Big City	14,688	0.16	0.37	14,000	0.35	0.48
Self employment						
Share with Business Inc.	14,688	0.0007	0.03	14,000	0.03	0.17
Business Inc.	10	361	357	405	433	457
Municipality characteristics						
Pop	14,688	67,561	104,069	14,000	129,632	141,317
# coethnics	14,688	403	936	14,000	1,163	1,738
Share of population	14,688	0.005	0.006	14,000	0.01	0.01
# coethnics with wage>0	14,688	200	497	14,000	315	525
# S-E coethnics	14,688	15.9	42.5	14,000	45.99	81.85
Share of population	14,688	0.0002	0.0003	14,000	0.0003	0.0005
Share of coethnics	14,688	0.025	0.038	14,000	0.039	0.043
$\geq$1 S-E coethnic	14,688	0.53	0.50	14,000	0.83	0.38

Notes: Big City implies staying in one of the three biggest cities, Stockholm, Malmö or Gothenburg. Share with Business Inc. shows the share with any positive declared (active) business income. Business Inc. is conditional on having some income from business activity. Income is given in hundreds of Swedish SEK (in 1990 \$1 $\approx$ 6 SEK). The education variables are measured two years after arrival due to poor quality the year of arrival. Municipality characteristics show information on municipality level. Hence # coethnics is the average number of coethnics in the municipality for a person in the sample. S-E coethnic shows the number of coethnics who are self-employed, measured as those who have any business income. $\geq$1 S-E coethnic is a dummy for the percentage in the sample that stays at a municipality with at least one self-employed coethnic.

Table A2: Top sectors of establishment and characteristics of the self-employed

Establishments	Freq	Percent	
Restaurants	146	23.89	
Retail sale in non-specialized stores	43	7.04	
Retail sale in tobacco store	31	5.07	
Hair and beauty services	21	3.44	
Taxi services	18	2.95	
Retail sale of fruits and vegetables	11	1.8	
Other	112	18.3	
Unknown	229	37.4	
Characteristics 90-91	Obs	Mean	Std. Dev.
Age	611	29.05	7.04
Married	611	0.43	0.50
Sex	611	0.88	0.32
Children	611	0.35	0.48
#Children (| parent)	215	2.13	1.25
Highly Educated	611	0.06	0.23
$\leq$ 9 years of education	611	0.81	0.39
Big city	611	0.15	0.36

Notes: Upper panel: Establishments for the self-employed, based on 4-digits sni-codes. The lower panel shows individual characteristics for the 611 self-employed in the sample.

B Auxiliary Analyses

A first robustness check is to consider a change in the dependent variable. So far, I used a definition relying on whether an individual had business income. Another way to capture self-employment would be to use a definition based on being registered as self-employed in 1995 or 1996 with the tax authorities, essentially implying self-employment as the main income source. I apply the latter definition in the estimations in Table B1, and as can be seen from the coefficients, the estimates remain almost the same as in the baseline case.

Second, in the baseline case, Table 2, I included two specifications, one without any covariates and fixed effects and one with all the controls from the preferred specification. In Table B2, I show several specifications, including different combinations of controls. In general, fixed effects for birth country are important for the coefficient's size, but the main estimates' size varies little over specifications. In the last column, I further add a quality control for birth country at the municipality of arrival, which is the employment rate for coethnics at municipality level. Clearly, the addition of a control for employment rate for coethnics does not matter for the coefficient size.

Table B3 widens the definition of enclave, using 1) an interaction term and 2) a broader base for ethnicity, using languages spoken in the birth country. The grouping of languages is seen in Table B4. Moreover, as an additional test, I further looked into effects using probit and logit estimations, which both give qualitatively similar results. These are found in Table B5.

Furthermore, the baseline case used in Table 2 standardizes the explanatory variables with the municipality population. In Table B6 I try out other functional

forms. First, to take into consideration any extreme values or decreasing returns to scale, I use a log transformation of my two main treatments. Note, however, that the share of self-employed coethnics entails a sizable share of zeros. I, therefore, used an inverse hyperbolic transformation: $ln(z+\sqrt{1+z^2})$. The transformation has the nice feature of sustaining all zeros as zeros, while creating a log-like interval for the numbers larger than 0. More specifically, the transformation keeps values $\approx$1 close to the original value, while approaching a log approximation as the value increases. If large outliers with many self-employed coethnics at arrival are driving the baseline results, I expect an estimation using an inverse hyperbolic sine function to show vastly different results. The effect is seen in column (1) and shows that the self-employed coethnics had a significant and positive effect, while no significant effect could be detected for the other coethnics. The second column of Table B6 shows that the same holds true for using the log of the absolute number of coethnics, as well as the log of the self-employment rate among coethnics in the municipality.

Table B1: Estimates changing the definition of the dependent variable, using definition from labor market survey rather than having business income or not

VARIABLES	(1)<br>Registered as<br>Self-employed	(2)<br>Registered as<br>Self-employed
# Self-employed Coethnics		
(As share of municipality population)	0.0109***	
	(0.00392)	
# Non-Self-employed Coethnics		
(As share of municipality population)	-0.00665**	
	(0.00309)	
# Self-employed Coethnics		
(Inverse Hyperbolic sine functional form)		0.00457**
		(0.00191)
# Non-Self-employed Coethnics		
(Inverse Hyperbolic sine functional form)		0.000534
		(0.00193)
Observations	12,461	13,909
Mean Dep. Variable	0.027	0.027
Covariates and Fixed Effects	YES	YES

Notes: Estimations changing the definition of the dependent variable, using definition from labor market survey rather than having business income or not. *** $p<0.01$, ** $p<0.05$, * $p<0.1$. Standard errors clustered on municipality level. See Table 2 for information on covariates.

Table B2: Baseline regression, including different sets of covariates and fixed effects

VARIABLES	(1)<br>S-E<br>or not	(2)<br>S-E<br>or not	(3)<br>S-E<br>or not	(4)<br>S-E<br>or not	(5)<br>S-E<br>or not	(6)<br>S-E<br>or not
# Self-employed Coethnics						
(As share of municipality pop)	0.0252***	0.0266***	0.0176***	0.0177***	0.0215***	0.0214***
	(0.00518)	(0.00517)	(0.00521)	(0.00519)	(0.00555)	(0.00553)
# Non-Self-employed Coethnics						
(As share of municipality pop)	-0.0162***	-0.0149***	-0.00976**	-0.0100***	-0.0121***	-0.0120***
	(0.00415)	(0.00415)	(0.00379)	(0.00378)	(0.00450)	(0.00448)
Observations	14,000	14,000	14,000	14,000	14,000	14,000
Ind Controls	NO	YES	YES	YES	YES	YES
Country FE	NO	NO	YES	NO	NO	NO
Cohort by Country FE	NO	NO	NO	YES	YES	YES
Municipality FE	NO	NO	NO	NO	YES	YES
Country group Controls	NO	NO	NO	NO	NO	YES

Notes: Baseline Regression estimated with different set of controls. Regarding covariates: on country level I include share of employed coethnics as share of total amount of coethnics. For notes on specification and variables see Table 2.

Table B3: Effect of ethnic enclaves on self-employment using (i) interactions and (ii) language-based enclaves

VARIABLES	(1)<br>S-E<br>or not	(2)<br>S-E<br>or not	(3)<br>S-E<br>or not
# Non-Self-employed coethnics	9.26e-06		
	(6.12e-06)		
# Self-employed coethnics	0.000471*		
	(0.000240)		
Interaction term	-1.24e-07*		
	(6.45e-08)		
Non-Self-employed language coethnics			
(as share of mun. pop.)		0.00512	-0.00770
		(0.00347)	(0.00490)
Self-employed language coethnics			
(as share of mun. pop.)			0.0180***
			(0.00914)
Observations	14,000	14,000	14,000
Mean Dep. Variable	0.044	0.044	0.044
Covariates and Fixed Effects	YES	YES	YES

Notes: In column (1) I interact the absolute number of the two explanatory variables. Column (2) and (3) replicate the baseline regressions (Table 2), only the definition of the enclave is based on languages instead of birth country. The partition of the language groups is described in Table B4. Note that column (2) includes only the use of all, non-self-employed coethnics as explanatory variable, and column (3) adds all self-employed coethnics. See Table 2 for more information on specification.

Table B4: Languages and country of birth groups

Language groups (in the sample)	Birth Country
Serbo-Croatian	Former Yugoslavia, Bosnia, Serbia, Croatia, Macedonia
Arabic	Somalia, Lebanon, Syria, Iraq, Tunisia, Morocco, Algeria, Egypt
Persian	Iran, Afghanistan
Amharic	Ethiopia
Romanian	Romania
Bulgarian	Bulgaria
Vietnamese	Vietnam

Table B5: Regressing having business income or not on the standardized share of self-employed coethnics and standardized share of coethnics, using Probit (column 1 and 2) and Logit models (column 3 and 4)

VARIABLES	(1)<br>S-E<br>or not<br>Odds Ratios	(2)<br>S-E<br>or not<br>Odds Ratios	(3)<br>S-E<br>or not<br>Odds Ratios	(4)<br>S-E<br>or not<br>Odds Ratios
# S-E Coethnics				
(As share of mun. pop.)	0.240***	0.233***	0.502***	0.482***
	(0.0457)	(0.0462)	(0.0992)	(0.0925)
# Non-S-E Coethnics				
(As share of mun. pop.)	-0.184***	-0.157***	-0.402***	-0.322**
	(0.0538)	(0.0653)	(0.119)	(0.136)
Observations	14,000	11,825	14,000	11,825
Covariates and FE	NO	YES	NO	YES

Notes: Probit and logit estimations. Coefficients represent odds ratios. See Table 2 for information on covariates and clustering.

Table B6: Redoing baseline regression using different functional forms

VARIABLES	(1)<br>S-E<br>or not	(2)<br>S-E<br>or not
$\ln \frac{SE-coethnics}{Pop}$	0.0130***	
	(0.00407)	
$\ln \frac{Coethnics}{Pop}$	-0.000492	
	(0.00280)	
$\ln \frac{SE-coethnics}{Coethnics}$		0.0111***
		(0.00372)
ln(Coethnics)		0.00693
		(0.00472)
Observations	14,000	14,000
Covariates and FE	YES	YES

Notes: Baseline regression using different functional forms. Column (1) transforms the number of self-employed coethnics as share of the population using an inverse hyperbolic sine transformation. For the second treatment, coethnics as share of population, I simply use a log transformation. Column (2) logs the absolute number of coethnics as well as the self-employment rate within the ethnic group.
